# *In Vivo* and *In Vitro* Protein Ligation by Naturally Occurring and Engineered Split DnaE Inteins

**DOI:** 10.1371/journal.pone.0005185

**Published:** 2009-04-13

**Authors:** A. Sesilja Aranko, Sara Züger, Edith Buchinger, Hideo Iwaï

**Affiliations:** Research Program in Structural Biology and Biophysics, Institute of Biotechnology, University of Helsinki, Helsinki, Finland; Swiss Federal Institute of Technology Lausanne, Switzerland

## Abstract

**Background:**

Protein *trans*-splicing by naturally occurring split DnaE inteins is used for protein ligation of foreign peptide fragments. In order to widen biotechnological applications of protein *trans*-splicing, it is highly desirable to have split inteins with shorter C-terminal fragments, which can be chemically synthesized.

**Principal Findings:**

We report the identification of new functional split sites in DnaE inteins from *Synechocystis sp*. PCC6803 and from *Nostoc punctiforme*. One of the newly engineered split intein bearing C-terminal 15 residues showed more robust protein *trans*-splicing activity than naturally occurring split DnaE inteins in a foreign context. During the course of our experiments, we found that protein ligation by protein *trans*-splicing depended not only on the splicing junction sequences, but also on the foreign extein sequences. Furthermore, we could classify the protein *trans*-splicing reactions in foreign contexts with a simple kinetic model into three groups according to their kinetic parameters in the presence of various reducing agents.

**Conclusion:**

The shorter C-intein of the newly engineered split intein could be a useful tool for biotechnological applications including protein modification, incorporation of chemical probes, and segmental isotopic labelling. Based on kinetic analysis of the protein splicing reactions, we propose a general strategy to improve ligation yields by protein *trans*-splicing, which could significantly enhance the applications of protein ligation by protein *trans*-splicing.

## Introduction

Protein splicing is a post-translational modification, in which an intervening protein splicing domain (intein) catalyzes ligation of the two flanking N- and C-terminal segments (N-extein and C-extein) by a peptide bond and concomitantly excises itself from the precursor protein [1]–[3]. Protein splicing can also take place in *trans* by ligating separate protein fragments containing each half of a naturally or artificially split intein (N-intein and C-intein) [4]–[6]. This protein *trans*-splicing (PTS) could also work in foreign contexts where the naturally occurring extein segments are replaced with other foreign protein sequences of interest. Therefore, protein *trans*-splicing can be used for ligation of polypeptide chains with a peptide bond for protein semi-synthesis, protein cyclization, segmental isotopic labelling, and site-specific protein modifications [7]–[12]. Protein *trans*-splicing has also been exploited to control protein functions in living organisms as a post-translational modification [13]–[15]. Thus, protein *trans*-splicing could be widely used in biotechnology and chemical biology [16].

Inteins usually consist of two domains, namely, a Hint domain and an endonuclease domain [3]. Since only the Hint domain is required for protein splicing, several inteins have been minimized by removing the endonuclease domain for biotechnological applications [17]–[19]. The Hint domain could be reduced to as small as 135 residues, which is presumably the minimal functional length [19]. Naturally occurring split inteins contain 102–111 residues for N-intein (Int_N_) and 35–36 residues for C-intein (Int_C_) [20]. Short functional fragments of inteins have been of special interest because they could be easily prepared by chemical synthesis [12] and widen applications of protein *trans*-splicing for chemical modifications and protein semi-synthesis [21]. The shortest fragment identified so far is the N-terminal 11 residues of *Synechocystis sp*. PCC6803 (*Ssp*) DnaB intein [22]. Our interest was to identify functional split DnaE inteins with a shorter C-intein. Shorter C-inteins could be used as a ligation tag that can be easily synthesized or fused with other proteins for protein ligation.

In this study, a series of split DnaE inteins with new split sites have been constructed and tested for protein ligation both *in vivo* and *in vitro* to identify a functional split DnaE intein with a minimal C-terminal fragment. The robustness of the short C-intein has been tested by ligation of two domains that could not be ligated by wild-type DnaE intein. We also investigated the effect of extein sequences on protein ligation by protein *trans*-splicing. The effect of various reducing agents on *in vitro* protein ligation was tested with several target proteins.

## Results

### Construction of split SspDnaE inteins with new split sites

**Figure 1:**
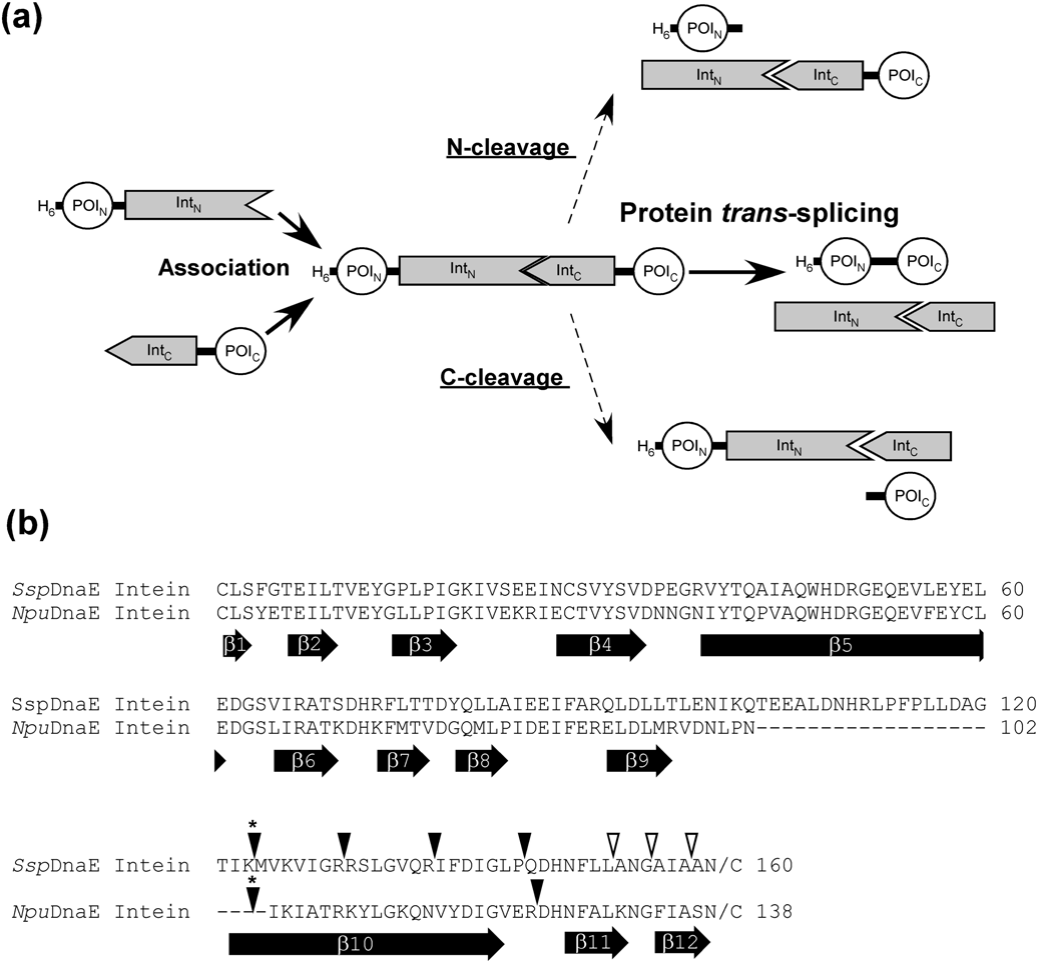
Protein *trans*-splicing and locations of the new split sites. (a) Schematic representation of the protein *trans*-splicing process and two possible side reactions of N- and C-cleavage. Two fragments of the protein of interest (POI) can be ligated by protein *trans*-splicing reaction. (b) Sequence alignment of *Ssp*DnaE and *Npu*DnaE inteins. The locations of the experimentally tested split sites of *Ssp*DnaE and *Npu*DnaE inteins are indicated by inverse triangles on the top of the primary sequences. The asterisks above inverse triangles indicate the naturally occurring split site. Filled triangles indicate the split sites, where the split inteins retained protein *trans*-splicing activity. Open triangles indicate the split sites, where no protein *trans*-splicing activity could be detected. The location of the b-strands observed in the crystal structures of *Ssp*DnaE intein (PDB code 1ZDE) [35] and *Ssp*DnaB mini-intein (1MI8) [36] are indicated at the bottom of the sequences. The numbering for b-strands is adapted from *Ssp*DnaB mini-intein [36].

*Ssp*DnaE intein is one of the naturally occurring split inteins widely used in biotechnological applications. Naturally occurring split inteins can spontaneously induce protein splicing in *trans* after association of the N- and C-terminal parts (Figure 1a). In contrast, artificially split inteins often require tedious denaturation and renaturation steps to restore protein splicing activity because of lower solubility of the precursor fragments [23]. Protein ligation of two flanking foreign sequences through protein *trans*-splicing by naturally split inteins usually requires no additional cofactor, but a few residues of the original extein sequences might be necessary for efficient splicing [11]. To identify new functional split inteins with a shorter C-intein, we have moved the split site in naturally split *Ssp*DnaE intein towards the C-terminus by shortening the C-terminal half (*Ssp*DnaE-Int_C_) systematically by 6–7 residues and elongating the N-terminal half (*Ssp*DnaE-Int_N_) by approximately the same lengths (Figure 1b, Figure S1). *Ssp*DnaE-Int_N_s were fused with the N-terminally His-tagged B1 domain of protein G (GB1), of which expression was under the control of an inducible T7 promoter [8]. Previously, we found that the change of the N-terminal junction sequence of EY from *Ssp*DnaE to other sequences such as GS had little influence on the ligation yield [24]. Therefore, we used a linker of GS originated from the restriction site of *Bam*HI between GB1 and *Ssp*DnaE-Int_N_s. *Ssp*DnaE-Int_C_s were also fused to a chitin binding domain (CBD), of which expression was controlled by an arabinose promoter (Figure S1). We kept the sequence of CFNK from the wild-type junction sequence of *Ssp*DnaE and added GT for the cloning site of *Kpn*I as a linker between *Ssp*DnaE-Int_C_s and CBD [8]. GB1 and CBD were used here as model proteins because they are small soluble proteins. The N- and C-precursor proteins were genetically encoded into two separate plasmids that bear the compatible RSF3010 and ColE1 origins [8]. Seven plasmids for each half were constructed for testing *in vivo* and *in vitro* protein ligation (Table S1 and Figure S1).

### *In vivo* protein *trans*-splicing by the new split *Ssp*DnaE inteins

**Figure 2:**
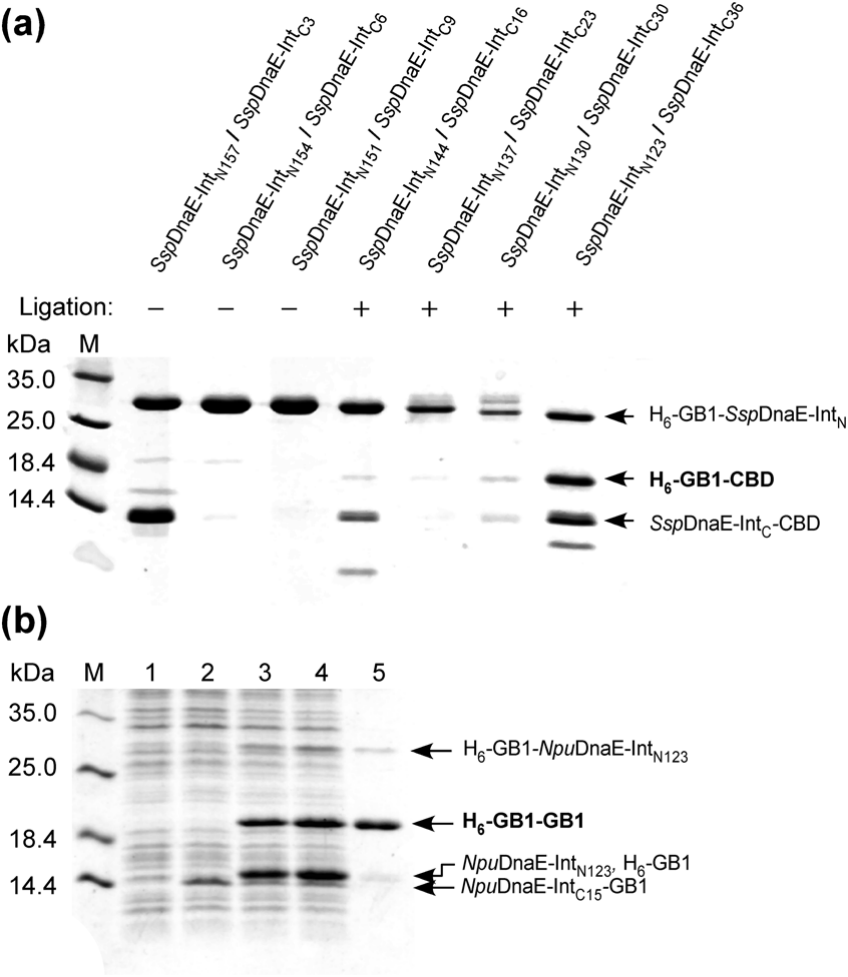
*In vivo* protein ligations by the newly engineered split *Ssp*DnaE and *Npu*DnaE inteins. (a) SDS-PAGE analysis of *in vivo* protein ligations by the newly engineered split *Ssp*DnaE inteins after purification with Ni-NTA. The combinations of *Ssp*DnaE-Int_N_ and *Ssp*DnaE-Int_C_ are indicated on the top of the lanes. (b) *In vivo* protein ligation by *Npu*DnaE intein with the newly engineered split site (*Npu*DnaE-Int_N123/C15_). Lane 1, before induction; lane 2, 1.5 hours after induction only with arabinose; lane 3, 1.5 hours after additional induction with IPTG; lane 4, 3 hours after induction with IPTG and arabinose; lane 5, elution from Ni-NTA column.

Protein ligation by the new split inteins was tested *in vivo* using the dual vector system previously developed in our group [8]. This system allows us to conveniently check protein ligation because protein ligation could be initiated by the induction of the two precursor fragments with the two inducers, isopropyl-β-D-thiogalactoside (IPTG) and arabinose, and subsequently analyzed by SDS-PAGE [24]. Moreover, endogenous auxiliary factors such as chaperones might improve protein ligation in cells by promoting correct protein folding. The C-terminal part was always first induced for 0.5 hours ensuring an excess of the C-terminal precursor prior to the expression of the N-terminal precursor, and followed by the induction of the N-terminal precursor for another 3.5–5.5 hours. The pre-existing C-terminal precursor protein could be converted into the ligated product through protein *trans*-splicing after the association with the N-terminal part and protein splicing. The expression level of the N-terminal fragment was monitored by SDS-PAGE in order to avoid an enormous excess of the N-terminal part, which could underestimate the ligation yields. Immobilized Metal Affinity Chromatography (IMAC) was used to purify the N-terminal His-tagged precursor, the ligated product, and, if any, the cleaved N-terminal GB1 produced by the side reactions (Figure 1a). If i*n vivo* protein ligation works with 100% efficiency and if there is no excess of the N-terminal precursor, only H_6_-GB1-CBD will be purified by IMAC through the N-terminal His-tag. If the N- and C-terminal fragments associate with each other but no protein splicing is induced, both N- and C-terminal fragments (H_6_-GB1-*Ssp*DnaE-Int_N_ and *Ssp*DnaE-Int_C_-CBD) will be purified owing to the affinity between them. Furthermore, if the N- and C-inteins do not interact or if the C-terminal cleavage reaction is the dominant reaction after association of the N- and C-inteins, a single band of the N-terminal precursor is expected to be visible in the SDS gel. In some cases, during protein purification and sample preparation for SDS-PAGE, reactions such as splicing and cleavages could take place, which produced smaller bands of cleaved and spliced products. The ligated product was confirmed by mass-spectrometry (Figure S2). We could identify the ligated product H_6_-GB1-CBD in the elution fractions from IMAC only for the combinations of *Ssp*DnaE-Int_N123_/*Ssp*DnaE-Int_C36_ (wild-type), *Ssp*DnaE-Int_N130_/*Ssp*DnaE-Int_C30_, *Ssp*DnaE-Int_N137_/*Ssp*DnaE-Int_C23_, and *Ssp*DnaE-Int_N144_/*Ssp*DnaE-Int_C16_ (Figure 2a). The ligation yields were estimated from the ratios between the intensities of the ligated product and one of the most abundant residual precursor fragments in the SDS gel, which were ca. 3% for *Ssp*DnaE-Int_N144_/*Ssp*DnaE-Int_C16_, ca. 1% for *Ssp*DnaE-Int_N137_/*Ssp*DnaE-Int_C23_, and ca. 16% for *Ssp*DnaE-Int_N130_/*Ssp*DnaE-Int_C30_. These efficiencies might be underestimated if an excess of the N-terminal part was present during the expression due to the co-purification of the N-terminal precursor containing an N-terminal His-tag. The highest yield was estimated for the wild-type combination of *Ssp*DnaE-Int_N123_/*Ssp*DnaE-Int_C36_ (67%). Albeit the amounts of the ligated products produced by the newly engineered inteins were very small, the protein ligation was still detectable by SDS-PAGE. The split site of *Ssp*DnaE-Int_N144_/*Ssp*DnaE-Int_C16_ was the split site of the shortest C-intein retaining detectable splicing activity. However, the ligation efficiency was significantly lower than that of wild-type *Ssp*DnaE intein because of the low splicing activity and the side reactions. The pairs of *Ssp*DnaE-Int_N151_/*Ssp*DnaE-Int_C9_ and *Ssp*DnaE-Int_N154_/*Ssp*DnaE-Int_C6_ could not induce protein *trans*-splicing as only the N-terminal precursor was purified, indicating there was no significant interaction between them. On the other hand, the shortest C-intein construct of *Ssp*DnaE-Int_C3_ was purified together with the N-terminal *Ssp*DnaE-Int_N157_ indicating that there was sufficient interaction between them. However, we could not identify any ligated product although there was a band at 18.4 kDa in the SDS gel indicating a small amount of the N-cleavage reaction that produced Int_N_.

### Split *Npu*DnaE intein with the new split site

The low ligation efficiencies of the newly functional split sites of *Ssp*DnaE intein suggest little practical use of these new split inteins. However, we have recently discovered that DnaE intein from *Nostoc punctiforme* (*Npu*) has more robust protein *trans*-splicing activity than that of *Ssp*DnaE intein and is also more tolerant of amino acid replacements at the C-terminal splicing junction [24]. Our previous study indicated that the N-terminal part (*Npu*DnaE-Int_N_) is responsible for the higher ligation efficiency [24]. Therefore, we were interested in introducing the new split site of *Ssp*DnaE intein into *Npu*DnaE intein to obtain sufficient protein *trans*-splicing activity for practical use. The new split site with the C-terminal 16 residues in *Ssp*DnaE is located between β-strands 10 and 11 (Figure 1b). We decided to shorten the C-intein by one more residue in *Npu*DnaE intein because based on the NMR structures of *Npu*DnaE intein (PDB entry, 2KEQ) the split site would be still in the loop between β-strands 10 and 11 [25], [26]. Protein ligation *in vivo* by *Npu*DnaE-Int_N123_/*Npu*DnaE-Int_C15_ is demonstrated in Figure 2b. The C-terminal part (*Npu*DnaE-Int_C15_-GB1) was induced first by l-arabinose (lane 2, Figure 2b). After the consecutive induction of the N-terminal part (H_6_-GB1-*Npu*DnaE-Int_N123_), a large amount of the ligated product (H_6_-GB1-GB1) was accumulated (lane 3 and 4, Figure 2b). The fraction purified by IMAC contained almost no precursor proteins and the ligation was confirmed by mass spectrometry (lane 5, Figure 2b and Figure S3). We estimated the ligation efficiency to be ca. 96%, which is significantly better than any of the tested combinations of the newly split *Ssp*DnaE inteins. We also tested protein ligation by the combination of *Npu*DnaE-Int_N123_/*Ssp*DnaE-Int_C16,_ which resulted in similar ligation efficiency (data not shown). This result emphasizes the dominant contribution of the N-intein to the ligation efficiency and suggests that the sequence variation between *Npu*DnaE-Int_C15_ and *Ssp*DnaE-Int_C16_ (the sequence identity is 66%) has little influence on protein *trans*-splicing efficiency.

### Protein ligation of SH3 domains by the naturally split *Npu*DnaE intein

**Figure 3:**
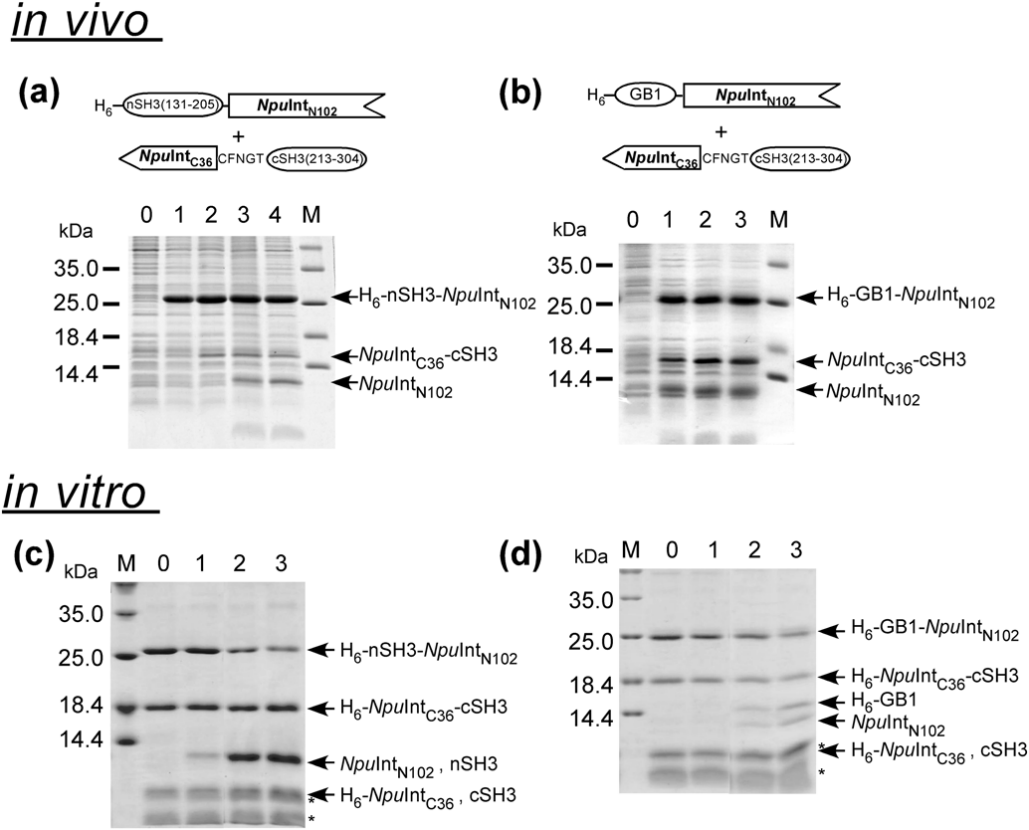
Protein ligation *in vivo* and *in vitro* by the naturally occurring split *Npu*DnaE intein. (a) Protein ligation of nSH3 and cSH3 *in vivo* by naturally occurring split *Npu*DnaE intein. Lane 0, before induction; lane 1, 1 hour after the induction with IPTG and arabinose; lane 2, 2 hours; lane 3, 4 hours; lane 4, 6 hours. (b) Protein ligation of GB1 and cSH3 *in vivo* by the wild-type *Npu*DnaE intein. Lane 0, before induction; lane 1, 2 hours after the induction with IPTG and arabinose; lane 2, 4 hours; lane 3, 6 hours. *In vitro* protein ligation (c) of nSH3 and cSH3 (d) of GB1 and cSH3 in the presence of 50 mM DTT. Lane 0, 0 min after the mixing; lane 1, 10 min; lane 2, 3 hours; lane 3, 24 hours for (c). Lane 0, 0 min after the mixing; lane 1, 3 min; lane 2, 3 hours; lane 3, 24 hours for (d). Asterisks indicating the bands below 14.4 kDa in (c) and (d) are impurities from the purification of H_6_-*Npu*Int_C36_-cSH3.

**Figure 4:**
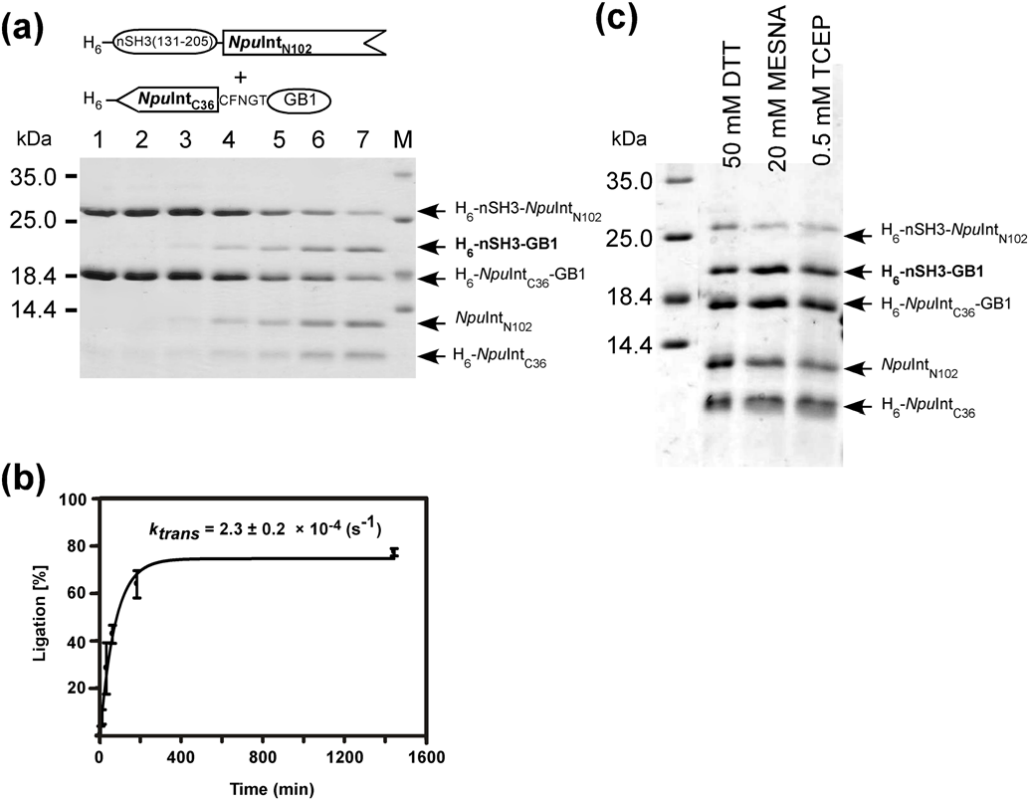
*In vitro* protein ligation of nSH3 and GB1 by the naturally occurring split *Npu*DnaE intein. (a) Time course of the protein ligation of nSH3 and GB1 by naturally occurring split *Npu*DnaE intein in the presence of 50 mM DTT. Lane 1, 0 min after the mixing; lane 2, 3 min; lane 3, 10 min; lane 4, 30 min; lane 5, 1 hour; lane 6, 3 hours; lane 7, 22 hours. (b) Kinetic analysis of the protein ligation from the SDS-PAGE. (c) SDS-PAGE analysis of the ligation reaction after overnight incubation in the presence of different reducing agents.

The robustness of naturally split *Npu*DnaE intein encouraged us to use *Npu*DnaE intein as a general tool for protein ligation and to apply it to biologically relevant proteins [24]. The Src homology 3 (SH3) domain is one of the most abundant domains in multi-domain proteins. Therefore, we were interested in protein ligation of the two SH3 domains from c-Crk-II adaptor protein [27]. Despite the robustness of *Npu*DnaE intein, protein ligation of the two SH3 domains by wild-type *Npu*DnaE intein was not possible, because the side reactions were dominating the *trans*-splicing and producing mainly cleaved products (Figure 3a and 3c). When the N-terminal SH3 (nSH3) was replaced with the model protein GB1, both *in vivo* and *in vitro* ligation of the two proteins by protein *trans*-splicing was still not possible with high yields (Figure 3b and 3d, Figure S4). On the other hand, the ligation of the two proteins *in vitro* as well as *in vivo* was significantly improved after replacing the C-terminal SH3 (cSH3) with GB1 (Figure 4a, Table 1). These observations indicate that protein *trans*-splicing can be significantly influenced not only by the sequences near the splicing junctions but also by the exteins, which brings additional complexity to protein *trans*-splicing. Furthermore, the replacement of the C-terminal precursor protein suggests that the C-terminal fragment containing cSH3 negatively affects the protein ligation.

**Table 1:** The final yields of the protein ligation by protein *trans*-splicing.

Intein	N-extein	C-extein	Yield (%) with 50 mM DTT	Yield (%) with 0.5 mM TCEP
*Npu*DnaE-Int_N102/C36_ (wild-type)	nSH3	cSH3	n.d.	n.d.
	GB1	cSH3	n.d.	n.d.
	nSH3	GB1	77±10	61±8
*Npu*DnaE-Int_N123/C15_	nSH3	cSH3	27±10	50±7
	Smt3	GB1	9±3	65±7

n.d., not detectable.

### The effect of reducing agent on *trans*-splicing

In theory, protein *trans*-splicing does not require any thiol agents for the reaction [3]. However, both N- and C-inteins of *Npu*DnaE intein contain unpaired cysteine residues that could form intermolecular disulfide bonds and they may prevent the appropriate association of the two fragments. Therefore, it is desirable to keep the reaction under reducing conditions with a sulfhydryl reductant. In a previous study on *Ssp*DnaE intein, it has been reported that the presence of 50 mM dithiothreitol (DTT) would almost totally block protein *trans*-splicing and instead shunt the reaction to *trans*-cleavage [28]. As a sulfhydryl reductant, we have tested two thiol agents (DTT and 2-mercaptoethane sulfonic acid, MESNA) and a trialkylphosphine (tris(2-carboxyethyl)phosphine, TCEP) that is unreactive with thiol groups such as cysteine (Figure 4c). In contrast to the previous report, we found that the effect of various reducing agents on protein ligation was negligible for the ligation between nSH3 and GB1 (Figure 4c) as well as for the ligation of the two SH3 domains (data not shown). In the case of nSH3 and cSH3, the reaction was always dominated by *trans*-cleavage rather than *trans*-splicing (Table 1, Figure 3). It was not possible to improve the ligation of those SH3 domains by replacing the reducing agent. For the ligation of nSH3 and GB1, *trans*-splicing was always observed regardless of the reducing agents used (Table 1, Figure 4c). To understand these puzzling effects, we analyzed the kinetics of the protein ligation. It is well accepted that protein-splicing reaction involves the four concerted steps: (1) N-S acyl shift, (2) *trans*-thioesterification, (3) Asn cyclization, and (4) S-N acyl shift, and possibly undesired side reactions of N- and C-cleavage (Figure 1a) [29]. The detailed kinetics of the individual steps has been previously characterized for *Ssp*DnaE intein [28]. We decided to approximate the reactions with a simple kinetic model as depicted in (I), in which the entire reaction was divided into the two parallel reactions: *trans*-splicing and cleavage reactions because the two reactions are both irreversible processes. In this model, we also assume that the formation of the precursor complex is fast relative to the subsequent reaction steps and the dissociation constant is much smaller than the protein concentration used in the experiments [28].
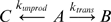
(I)


*A* = precursor complex, *B* = ligated product, *C* = cleaved product, *k_trans_* = 1^st^ order kinetic constant for *trans*-splicing, and *k_unprod_* = apparent 1^st^ order kinetic constant for all unproductive side reactions including the N- and C-terminal cleavage reactions. Time courses of the products can be formulated by the following rate equations.
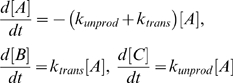
(II)


These equations can be easily solved [30]. The yield of the ligation at an infinite time can be derived from the two kinetic constants for *trans*-splicing and cleavage according to Eq. (III).
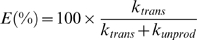
(III)


With this model, we should be able to estimate ligation yields from the rate constants of *trans*-splicing and side reactions, and *vice versa*. For the ligation between nSH3 and GB1, 2.3±0.2×10^−4^ (s^−1^) was estimated for *k_trans_* in the presence of 50 mM DTT (Figure 4b). According to Eq. (III) using the obtained kinetic constants and the reported DTT induced cleavage rate constant for *Ssp*DnaE intein (1.0±0.5×10^−3^ (s^−1^)) [28], the ligation yield for nSH3 and GB1 is expected to be 12–33%. However, the obtained final yield of close to 80% might suggest that the rate constant of DTT induced cleavage is about 1×10^−4^ with this system (Table 1). *Trans*-splicing was not detectable for nSH3 and cSH3, but the side reactions were dominant with the kinetic constant *k_unprod_* = 5.4±0.4×10^−4^ (s^−1^) in the presence of 50 mM DTT (data not shown). Although the replacement of DTT with TCEP as a reducing agent slowed the unproductive cleavage reactions, *trans*-splicing was not detectable. This suggests that *trans*-splicing reaction occurs at a significantly slower rate than the cleavage reaction. This model assumes that the association rates are fast and that the dissociation rates are similarly low for different exteins compared with the experimental concentration. Therefore, when the estimation of the yield is largely discrepant with the kinetic constants, the limiting factor is likely to be imposed by the association rate. Thus, this simple model and the kinetic analysis might provide a useful tool to predict final yields as well as to identify the rate-limiting step in protein *trans*-splicing reaction.

### Protein ligation by the newly engineered split *Npu*DnaE

**Figure 5:**
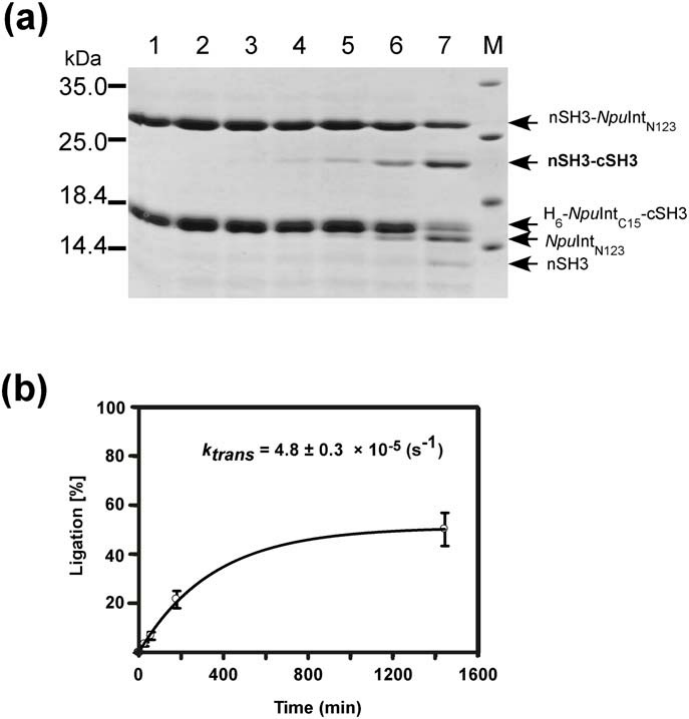
Protein ligation of two SH3 domains by the newly engineered split *Npu*DnaE intein. (a) SDS-PAGE analysis of the time course from the protein ligation reaction of nSH3 and cSH3 in the presence of 0.5 mM TCEP. Lane 1, 0 min after the mixing; lane 2, 3 min; lane 3, 10 min; lane 4, 30 min; lane 5, 1 hour; lane 6, 3 hours; lane 7, 22 hours. (b) Kinetic analysis of the protein ligation from the SDS-PAGE.

From the aforementioned results with the SH3 domains, we assumed that the C-intein fused with cSH3 is the limiting factor for the protein ligation of two SH3 domains, inducing fast cleavage reactions. We believe that cSH3 probably interferes with association of N- and C-inteins of wild-type *Npu*DnaE intein and that the shorter C-intein might not interfere the ligation of the two SH3 domains. Therefore, we decided to replace the intein with the newly engineered *Npu*DnaE intein (*Npu*DnaE-Int_N123_/*Npu*DnaE-Int_C15_) for the ligation. As demonstrated in Figure 5, the new split *Npu*DnaE intein could indeed ligate nSH3 and cSH3 that were not possible to be ligated by the naturally occurring split *Npu*DnaE intein. It demonstrates the effectiveness of the shorter C-intein in the case of difficult ligations such as the one between the two SH3 domains. The kinetic constants for *trans*-splicing were estimated to be 4.8±0.3×10^−5^ (s^−1^) in the presence of 0.5 mM TCEP (Figure 5b). Thus, the engineered split *Npu*DnaE intein can significantly improve the protein ligation by accelerating the *trans*-splicing reaction.

### Protein ligation of Smt3 and GB1

**Figure 6:**
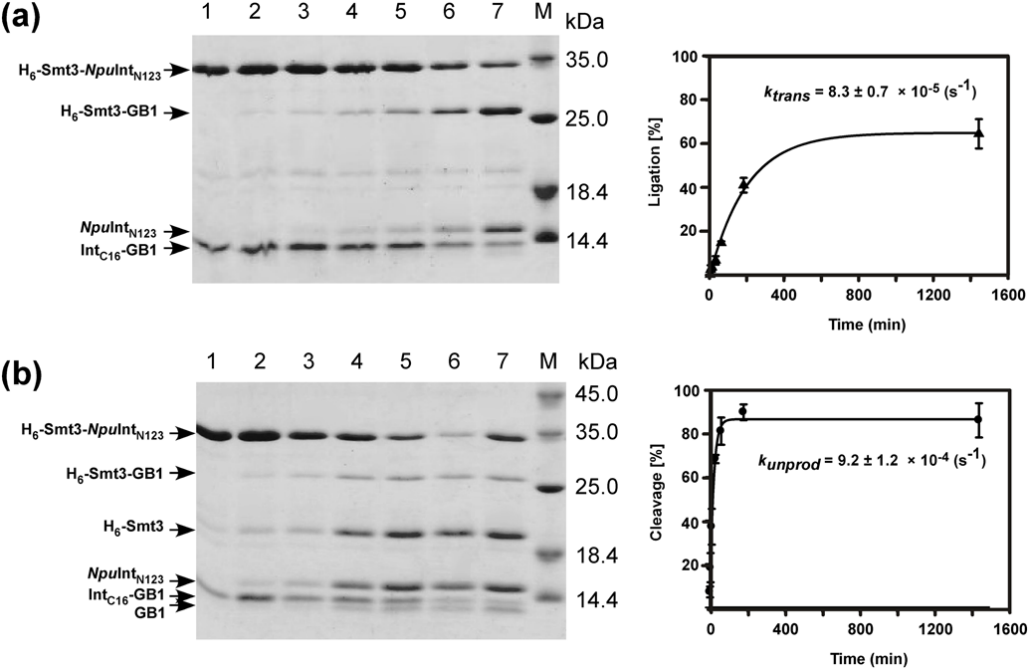
Protein ligation of Smt3 and GB1 by the newly engineered split *Npu*DnaE intein. Time courses and kinetic analysis of protein ligation in the presence of (a) 0.5 mM TCEP or (b) 50 mM DTT. SDS-PAGE: lane 1, 0 min after the mixing; lane 2, 3 min; lane 3, 10 min; lane 4, 30 min; lane 5, 1 hour; lane 6, 3 hours; lane 7, 24 hours.

Because of the strong influence of the extein sequences on protein *trans*-splicing, we wanted to test another small protein with a similar size, the yeast ubiquitin-like protein Smt3, for protein ligation [31]. Protein ligation of His-tagged Smt3 and GB1 by *Npu*DnaE-Int_N123_ was tested in the presence of either 50 mM DTT or 0.5 mM TCEP. The protein ligation of Smt3 and GB1 responded differently to the two different reducing agents. When 0.5 mM TCEP was used, the yield was more than 60–70%. On the contrary, only about 10% of the protein ligation was achieved in the presence of 50 mM DTT, where the cleavage reaction dominated. In this case the kinetic constant for *trans*-splicing in presence of 0.5 mM TCEP was estimated to be 8.3±0.7×10^−5^ (s^−1^). DTT induced the dominant cleavage reaction with a kinetic constant of 9.2±1.2×10^−4^ (s^−1^) (Figure 6). The protein ligation yield in the presence of 50 mM DTT is expected to be around 10% as it can be derived from Eq. (III) with an assumption that *trans*-splicing rates are similar for both DTT and TCEP. This is in good agreement with the yield obtained experimentally, suggesting that the simple model is appropriate for roughly estimating the yield without any intricate methods.

## Discussion

In this article, we demonstrated that C-intein from *Ssp*DnaE and *Npu*DnaE inteins could be shortened to C-terminal 16 or 15 residues without abolishing protein *trans*-splicing activity. The newly engineered split *Npu*DnaE intein bearing the C-terminal 15 residues as C-intein retained robust protein *trans*-splicing activity. The use of the shorter C-intein was even more effective for the ligation of the two SH3 domains that could not be ligated by the wild-type split DnaE inteins. The shorter length of C-intein of the engineered split *Npu*DnaE intein could be attractive for chemical synthesis and suitable for incorporation of chemically modified peptides by protein *trans*-splicing [32]. Moreover, the kinetic analysis of the ligation reaction could be important because the kinetic parameters are the key factor determining the ligation yields. The analysis using a simple parallel model to approximate the reaction could be a convenient tool to investigate the rate-limiting steps in the reaction and to estimate the ligation yields based on the kinetic parameters. Protein *trans*-splicing reaction in foreign contexts can be categorized into three groups. In the first group only side reactions of cleavages can be observed. Various reducing agents such as TCEP or DTT have little effect on improving protein ligation in this group. In this case, the cleavage reaction has a typical kinetic constant of >1×10^−4^ (s^−1^) and the *trans*-splicing rate is much slower than the cleavage rate. In the second group, regardless of the used reducing agents, protein ligation by protein *trans*-splicing can be observed. Here, the *trans*-splicing reaction is faster (>1×10^−4^ (s^−1^)) than the unproductive cleavage reactions induced by various reducing agents. In the third group, *trans*-splicing reaction is slower than the side reactions induced by DTT, but faster than the side reactions in the presence of TCEP. Therefore, the reducing agent could greatly influence the final yield. This is presumably because the thiol group of DTT is a nucleophile competing with the thiol of the first cysteine of C-intein and induces dominant cleavage reactions. However, the side reactions in the presence of TCEP are usually slower because it has no thiol group that functions as nucleophile competing with *trans*-splicing reaction.

In summary, we could create new functional split inteins with shorter C-inteins, which retained *trans*-splicing activity. Protein *trans*-splicing was found to be dependent on the protein sequences of the exteins even if the sequence around the splicing junctions were identical. How the exteins influence protein *trans*-splicing remains unclear. However, monitoring the kinetics of the protein *trans*-splicing reaction could be a useful tool to identify the rate-limiting steps in protein ligation reaction. To achieve a higher yield of protein ligation by protein *trans*-splicing, it is of importance to keep the competing side reactions slower than the *trans*-splicing reaction by replacing the reducing agent with non-thiol reducing agents such as TCEP or by accelerating the *trans*-splicing reaction using a more efficient split intein. The ligation between self-contained domains by protein *trans*-splicing was investigated in this article. However, the model describing the relation between *trans*-splicing and side reactions should be generally applicable even for the ligation within a single domain although such ligation may require refolding of the precursors which could be a more dominant factor affecting the yield. Further understanding of the factors influencing protein *trans*-splicing reaction rates such as folding processes of split inteins and engineering of split inteins could make protein *trans*-splicing a more versatile tool for protein modification, protein semi-synthesis, and segmental isotopic labelling.

## Materials and Methods

### Construction of plasmids

The N-terminal fragments of *Ssp*DnaE intein (*Ssp*DnaE-Int_N_) of various lengths were previously constructed [24]. The coding sequences of *Ssp*DnaE-Int_N_s were subcloned into pJJDuet30 between *Bam*HI and *Hin*dIII sites [8], resulting in the sequences coding for H_6_-GB1-*Ssp*DnaE-Int_N_s (Figure. S1). *Ssp*DnaE-Int_C_s of various lengths were constructed from the plasmid pSZRS1 containing the gene of the full-length *Ssp*DnaE-Int_C_ and the chitin binding domain (CBD) using synthetic oligonucleotides (Table S1) and cloned into pBAD vector (Figure S1).

The plasmid for H_6_-GB1-*Npu*DnaE-Int_N123_ was constructed by replacing the codon of residue 124 of the full-length *Npu*DnaE intein with a stop codon in the plasmid of pSKDuet16, resulting in pHYDuet36 [26]. The plasmid pSKDuet16 contains an additional two mutations of HM to LG at the front of GB1 due to the replacement of *Nde*I site by *Avr*II site, compared with the plasmids derived from pJJDuet30 [8]. The gene of *Npu*DnaE-Int_C15_-GB1 was amplified from pSKDuet16 and cloned into a pBAD vector (pHYBAD44) [24]. *Npu*DnaE-Int_N123_ was subcloned from pHYDuet36 into pHYRSF53LA using *Bam*HI and *Hin*dIII sites, which resulted in pHYRSF53-36 coding for H_6_-Smt3-*Npu*DnaE-Int_N123_ [26].

The gene of the N-terminal SH3 domain was amplified from pAT044 [33] with the two oligonucleotides (#HK009 and #SK202) and cloned into pHYRSF1-12 using *Nde*I and *Ahd*I sites, which resulted in pTMRSF07 (Table S1). The plasmid pHYRSF1-12 was previously constructed by transferring the gene of GB1 and the N-terminal *Npu*DnaE from pSKDuet1 into pRSF-1b using *Nco*I and *Hin*dIII sites. The plasmid of pHYRSF1-12 contains additional mutations of GS to TK to introduce *Ahd*I site at the front of *Npu*DnaE intein. The gene of the C-terminal SH3 domain was amplified from pAT044 with the two oligonucleotides (#SK199 and #SK200) and cloned into pSKBAD2 using *Kpn*I and *Hin*dIII sites (pHYBAD2-03) (Table S1). The plasmid pSARSF03, which codes for H_6_-*Npu*DnaE-Int_C36_-cSH3, was constructed by subcloning the genes of Int_C36_ and cSH3 into pHYRSF1-2 by *Nde*I and *Hin*dIII sites. The plasmids (pMMRSF17 for nSH3 and pMMRSF1-16 for cSH3) coding for the SH3 domains fused to the newly designed split *Npu*DnaE intein were previously described [34].

### Expression and purification of new split inteins

His-tagged DnaE-Int_N_s fused with GB1, Smt3, or SH3 domains were purified using His-Trap columns (GE Healthcare) under native condition. The DnaE-Int_C_s without a His-tag fused with GB1 were purified with IgG sepharose (GE Healthcare) according to the manufacturer's protocol. The eluted fractions of DnaE-Int_C_s were dialyzed against 10 mM Tris, 500 mM NaCl, 1 mM EDTA, pH 7.0 prior to protein ligation.

### *In vivo* protein ligation

Each pair of the two plasmids encoding N- or C-terminal precursor proteins was transformed into *E.coli* ER2566 (New England Biolabs) for protein expression. The cells bearing these two plasmids were grown in 25 ml LB medium supplemented with 100 µg/ml ampicillin and 25 µg/ml kanamycin. The plasmid containing DnaE-Int_C_ was first induced for 0.5 hours at a final concentration of 0.04% arabinose when the cell density reached OD_600_ = 0.5–0.8, followed by an additional induction of the N-terminal part with addition of a final concentration of 1 mM isopropyl-β-D-thiogalactoside (IPTG). Expression was carried out for another 4–5.5 hours. The cells were spun down at 4,500×*g* for 10 min and stored at −20°C for further purification. The harvested cells were lysed by ultrasonication in lysis buffer (50 mM sodium phosphate, 300 mM NaCl, 10 mM imidazole, pH 8.0). The cell debris was removed from the protein solution by centrifugation for 15 min at 18,000×*g*. The entire amount of the supernatant was loaded on a Ni-NTA spin column (Qiagen) equilibrated with lysis buffer and centrifuged for 2 min at 700×*g*. The column was washed twice with 600 µl washing buffer (50 mM sodium phosphate, 300 mM NaCl, 30 mM imidazole, pH 8.0). The bound protein was eluted from the spin column by washing twice with 200 µl elution buffer (50 mM sodium phosphate, 300 mM NaCl, 250 mM imidazole, pH 8.0).

### *In vitro* protein ligation

Equal amounts of the two precursor fragments (final concentrations of 15 µM) were mixed in the presence of final concentrations of 1 mM EDTA and either 50 mM DTT (dithiothreitol), 20 mM MESNA (2-mercaptoethane sulfonic acid), or 0.5 mM TCEP (tris(2-carboxyethyl)phosphine). The reactions were incubated at 25°C with shaking. The samples for SDS-PAGE analysis were typically taken at 0 min, 3 min, 10 min, 30 min, 1 hour, 3 hours, and 24 hours after mixing. The reactions were stopped by adding an equal amount of 1× SDS sample buffer containing 2-mercaptoethanol and stored at −20°C for over night. The samples were loaded on 18% SDS polyacrylamide gels after the incubation at 95°C for 5 min. The ligation yields were estimated from the intensities of the bands in the SDS-gels colored with Coomassie brilliant blue (PhastGel Blue R, GE Healthcare) by quantifying the scanned gels with ImageJ (NIH). The amounts of proteins were calculated with the assumption that the staining dye binds to the proteins equally. The errors were estimated by at least three independent reactions.

## Supporting Information

Table S1 List of the used oligonucleotides
(0.06 MB DOC)

Figure S1 The summary of the constructs for the newly engineered split SspDnaE inteins
(0.01 MB PDF)

Figure S2 The mass spectrum of the elution fraction from In vivo ligation of GB1 and CBD by SspDnaE intein.
(0.17 MB PDF)

Figure S3 The mass spectrum of the ligated product, H6-GB1-GB1 by the newly engineered NpuDnaE intein.
(0.05 MB PDF)

Figure S4 The mass spectra of the ligated and cleaved products from the ligation of nSH3 and GB1 by NpuDnaE intein.
(0.34 MB PDF)
