# Supplementary material for: In Vivo and In Vitro Protein Ligation by Naturally Occurring and Engineered Split DnaE Inteins
Source: PLoS One. 2009 Apr 13;4(4):e5185. doi: 10.1371/journal.pone.0005185 (PMC2664965; doi:10.1371/journal.pone.0005185)
Supplement: Table S1 — List of the used oligonucleotides (0.06 MB DOC) [file pone.0005185.s001.doc]

# Supplementary Table: List of the used oligonucleotides

| Plasmid | Gene | oligonucleotides |
| --- | --- | --- |
| pSZBAD09PG | *Ssp*DnaE-Int_C3_ | #SZ007:TGAATTTCATATGGCCAACTGTTTTAACAAAGG  #T7_term: CGTTTAGAGGCCCCAAGGGG |
| pSZBAD10PG | *Ssp*DnaE-Int_C6_ | #SK038: AATCATATGGCTATCGCCGCCAACTG  #T7_term: CGTTTAGAGGCCCCAAGGGG |
| pSZBAD08PG | *Ssp*DnaE-Int_C9_ | #SZ006: TGGATTTCATATGGCTAATGGTGCTATCG  #T7_term: CGTTTAGAGGCCCCAAGGGG |
| pSZBAD07PG, | *Ssp*DnaE-Int_C16_ | #SZ005: TGACTTTCATATGCAAGACCATAATTTTCTGC  #T7_term: CGTTTAGAGGCCCCAAGGGG |
| pSZBAD06PG | *Ssp*DnaE-Int_C23_ | #SZ004: TGAATTGCATATGATCTTTGATATCGGTCTGC  #T7_term: CGTTTAGAGGCCCCAAGGGG |
| pSZBAD05PG | *Ssp*DnaE-Int_C30_ | #SZ003:TGAATTTCATATGCGATCCCTGGGTGTGC  #T7_term: CGTTTAGAGGCCCCAAGGGG |
| pSZBAD01PG | *Ssp*DnaE-Int_C36_ | #SZ001:TGAATTTCATATGGTTAAAGTTATCG  #SZ002:TTGGGTACCTTTGTTAAAACAGTTGGC |
| pHYBAD44 | *Npu*DnaE-Int_C15_ | #HK146: TACATATGGACCATAATTTTGCACTC  #T7_term: CGTTTAGAGGCCCCAAGGGG |
| pTMRSF07 | nSH3 | #HK009: 5’CTTCCTGGTTACCTCCAATC  #SK202: 5’TCATATGCAGGAGGAGGCAGAGTATGTG |
| pHYBAD2-03 | cSH3 | #SK199: TTGGTACCCTGGGTGGGCCGGAGCCTG  #SK200: CGCAAGCTTAGCTGAAGTCCTCATCGGGATTC |
