# Supplementary material for: In Vivo and In Vitro Protein Ligation by Naturally Occurring and Engineered Split DnaE Inteins
Source: PLoS One. 2009 Apr 13;4(4):e5185. doi: 10.1371/journal.pone.0005185 (PMC2664965; doi:10.1371/journal.pone.0005185)
Supplement: Figure S1 — The summary of the constructs for the newly engineered split SspDnaE inteins (0.01 MB PDF) [file pone.0005185.s002.pdf]

Supplementary Figure 1

The summary of the constructs for the newly engineered split *Ssp*DnaE inteins

(a) Int_N constructs

*Ssp*DnaE-Int_{N157}	(pTTDuet19)	H_6-GB1 or H_6	-GS	*Ssp*$DnaE_N$(1-123)	-MVKVIGRRSLGVQRIFDIGLPQDHNFLLANGAIA
*Ssp*DnaE-Int_{N154}	(pSZDuet03)	H_6-GB1 or H_6	-GS	*Ssp*$DnaE_N$(1-123)	-MVKVIGRRSLGVQRIFDIGLPQDHNFLLANG
*Ssp*DnaE-Int_{N151}	(pTTDuet18)	H_6-GB1 or H_6	-GS	*Ssp*$DnaE_N$(1-123)	-MVKVIGRRSLGVQRIFDIGLPQDHNFLL
*Ssp*DnaE-Int_{N144}	(pTTDuet17)	H_6-GB1 or H_6	-GS	*Ssp*$DnaE_N$(1-123)	-MVKVIGRRSLGVQRIFDIGLP
*Ssp*DnaE-Int_{N137}	(pTTDuet15)	H_6-GB1 or H_6	-GS	*Ssp*$DnaE_N$(1-123)	-MVKVIGRRSLGVQR
*Ssp*DnaE-Int_{N130}	(pTTDuet05)	H_6-GB1 or H_6	-GS	*Ssp*$DnaE_N$(1-123)	-MVKVIGR
*Ssp*DnaE-Int_{N123}	(pTTDuet02)	H_6-GB1 or H_6	-GS	*Ssp*$DnaE_N$(1-123)	

(b) Int_C constructs

*Ssp*DnaE-Int_{C3}	(pSZBAD09)	MANCFNKGT-	CBD
*Ssp*DnaE-Int_{C6}	(pSZBAD10)	MAIAANCFNKGT-	CBD
*Ssp*DnaE-Int_{C9}	(pSZBAD08)	MANGAIAANCFNKGT-	CBD
*Ssp*DnaE-Int_{C16}	(pSZBAD07)	MQDHNFLLANGAIAANCFNKGT-	CBD
*Ssp*DnaE-Int_{C23}	(pSZBAD06)	MIFDIGLPQDHNFLLANGAIAANCFNKGT-	CBD
*Ssp*DnaE-Int_{C30}	(pSZBAD05)	MRSLGVQRIFDIGLPQDHNFLLANGAIAANCFNKGT-	CBD
*Ssp*DnaE-Int_{C36}	(pSZBAD01)	MVKVIGRRSLGVQRIFDIGLPQDHNFLLANGAIAANCFNKGT-	CBD
