# Supplementary material for: In Vivo and In Vitro Protein Ligation by Naturally Occurring and Engineered Split DnaE Inteins
Source: PLoS One. 2009 Apr 13;4(4):e5185. doi: 10.1371/journal.pone.0005185 (PMC2664965; doi:10.1371/journal.pone.0005185)
Supplement: Figure S2 — The mass spectrum of the elution fraction from In vivo ligation of GB1 and CBD by SspDnaE intein. (0.17 MB PDF) [file pone.0005185.s003.pdf]

Supplementary Figure 2

The mass spectrum of the elution fraction from *In vivo* ligation of GB1 and CBD by *Ssp*DnaE intein.

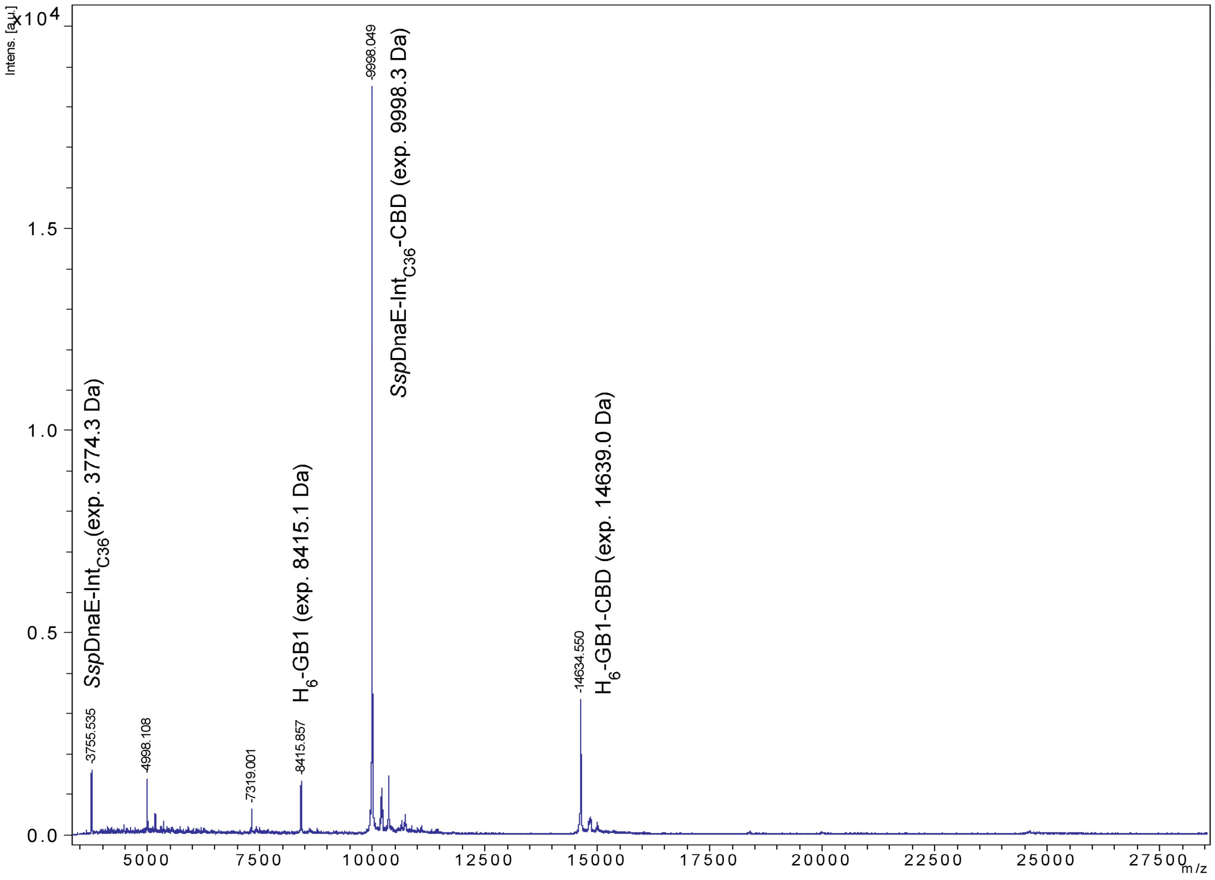

Supplemental Fig.2
