# Supplementary material for: In Vivo and In Vitro Protein Ligation by Naturally Occurring and Engineered Split DnaE Inteins
Source: PLoS One. 2009 Apr 13;4(4):e5185. doi: 10.1371/journal.pone.0005185 (PMC2664965; doi:10.1371/journal.pone.0005185)

Supplementary Figure 3

The mass spectrum of the ligated product, H_6-GB1-GB1 by the newly engineered *Npu*DnaE intein.

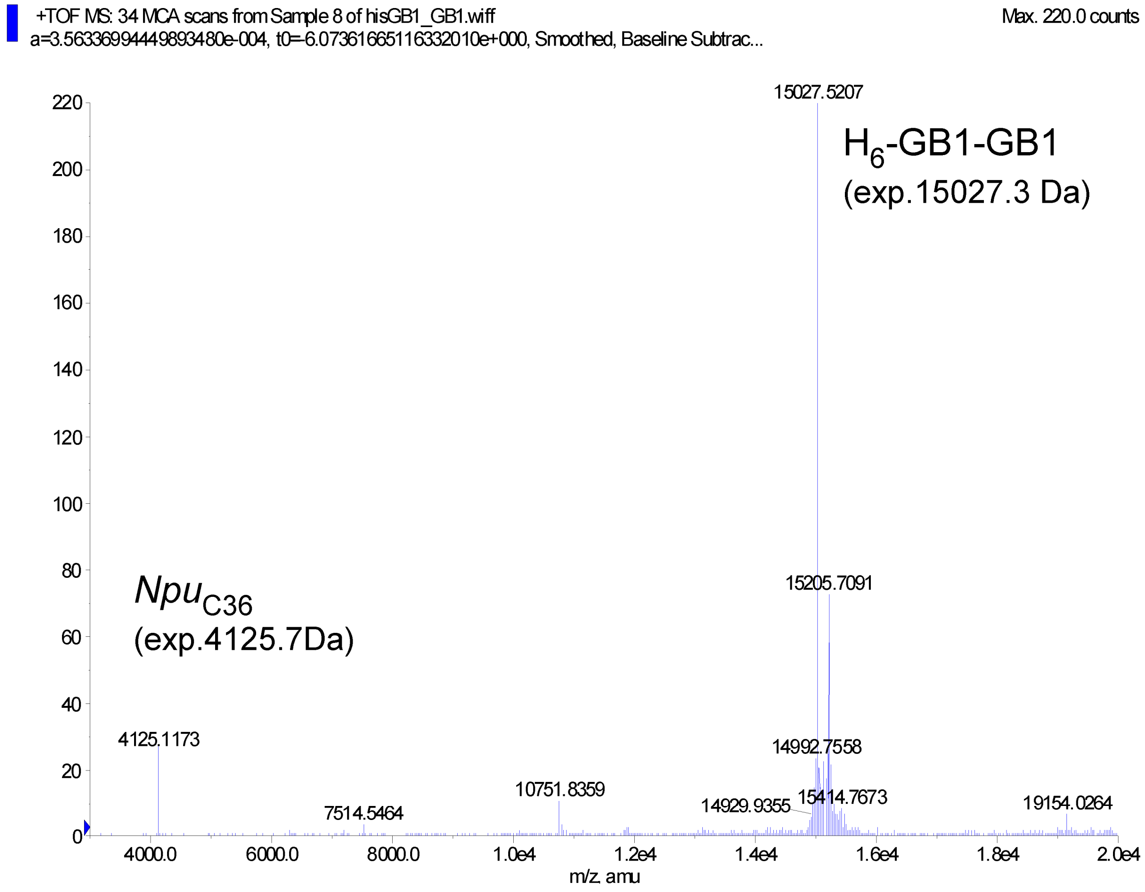

Supplement: Figure S3 — The mass spectrum of the ligated product, H6-GB1-GB1 by the newly engineered NpuDnaE intein. (0.05 MB PDF) [file pone.0005185.s004.pdf]
