# Supplementary material for: In Vivo and In Vitro Protein Ligation by Naturally Occurring and Engineered Split DnaE Inteins
Source: PLoS One. 2009 Apr 13;4(4):e5185. doi: 10.1371/journal.pone.0005185 (PMC2664965; doi:10.1371/journal.pone.0005185)
Supplement: Figure S4 — The mass spectra of the ligated and cleaved products from the ligation of nSH3 and GB1 by NpuDnaE intein. (0.34 MB PDF) [file pone.0005185.s005.pdf]

Supplementary Figure 4

The mass spectra of the ligated and cleaved products from the ligation of nSH3 and GB1 by *Npu*DnaE intein.

(A)

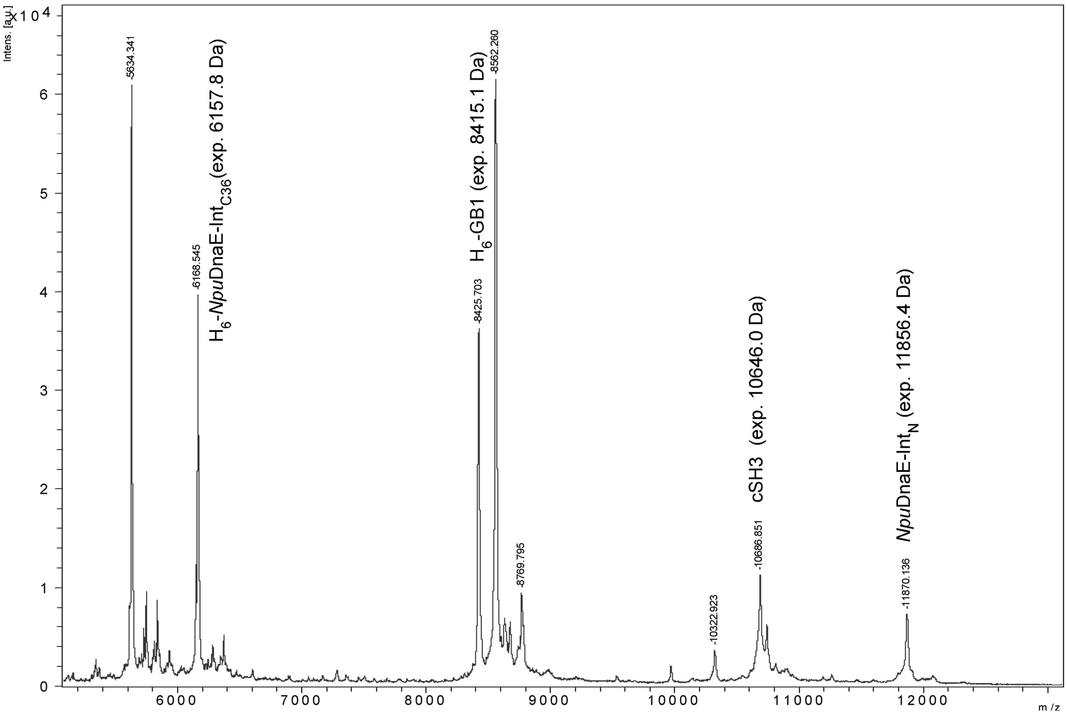

(B)

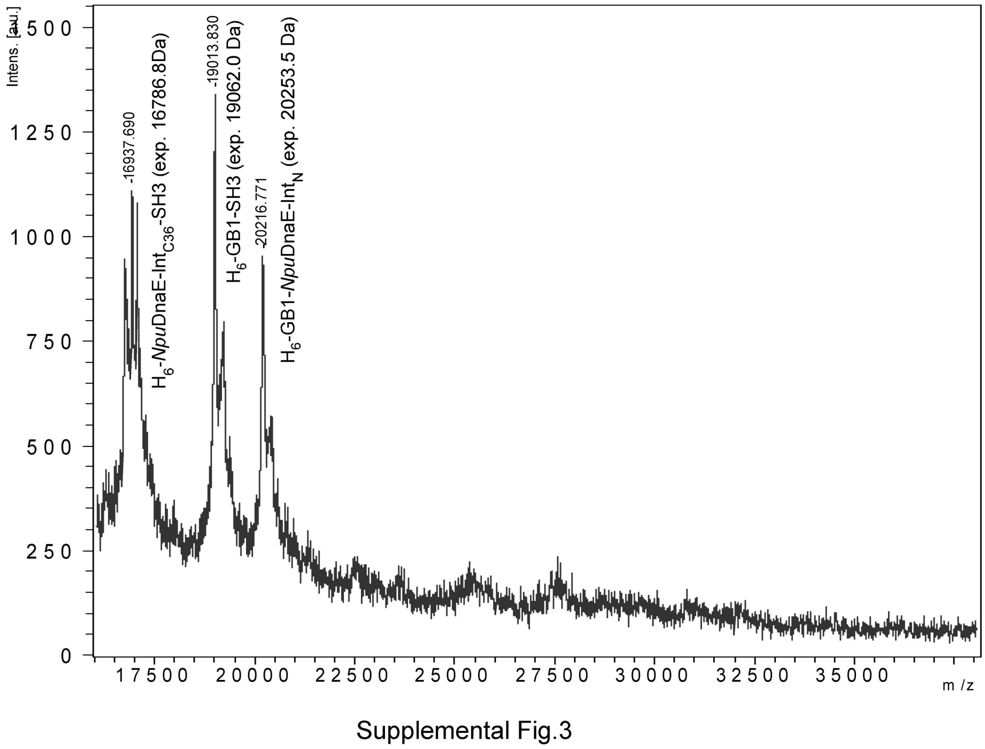

Supplemental Fig.3
